# Comparative Transcriptomic Analyses of Anthocyanin Biosynthesis Genes in Eggplant Under Low Temperature and Weak Light

**DOI:** 10.3390/plants14030478

**Published:** 2025-02-06

**Authors:** Baoying Shen, Hongqi Wu, Xinxin Xie, Bo Zhao, Peiqiang Chen, Deyong Ao, Heli Pan, Biying Lin

**Affiliations:** 1 Key Laboratory of Ministry of Education for Genetics, Breeding and Multiple Utilization of Crops, College of Horticulture, Fujian Agriculture and Forestry University, Fuzhou 350002, China; shenby889@foxmail.com (B.S.);; 2 Fuzhou Institute of Vegetable Sciences, Fuzhou 350002, China; xgsb2008@163.com

**Keywords:** eggplant (*Solanum melongena* L.), anthocyanin, transcriptome, transcription factor

## Abstract

Low temperature, weak light, and the combination of low temperature and weak light can have an impact on the growth, development, and quality of eggplants. The color of the eggplant peel is affected by the anthocyanin content. To better understand the influence of low temperature, weak light, and the combination of low temperature and weak light on the regulation of anthocyanins in the eggplant peel, four treatments were carried out on the eggplants, respectively: low temperature (18/13 °C, 250 μmol/(m^2^·s)), weak light intensity (WL, 25/20 °C, 120 μmol/(m^2^·s)), low temperature combined with weak light intensity (LW, 18/13 °C, 120 μmol/(m^2^·s)), and the control (CK, 25/20 °C, 250 μmol/(m^2^·s)). The effects of low temperature and weak light on the anthocyanin content in various parts of the eggplant were analyzed, and transcriptome analysis was performed on the eggplant peel under the treatments of low temperature, weak light, and the combination of low temperature and weak light using RNA sequencing. The anthocyanin content in eggplants increased under low temperature and the combination of low temperature and weak light treatments, while it decreased under weak light. KEGG analysis showed that three pathways, namely phenylpropanoid biosynthesis, flavonoid biosynthesis, and anthocyanin biosynthesis, were involved in the anthocyanin biosynthesis of eggplants. Pearson correlation coefficients indicated that the anthocyanin content in the eggplant peel under low temperature and the combination of low-temperature and weak-light treatments was significantly correlated with *SmPAL*, *Sm4CL*, *SmCYP73A100*, *SmCHS*, *SmCHI*, *F3H*, *DFR*, *ANS*, and *3GT*, and also significantly correlated with *MYB*, *bHLH*, and *AP2/ERF*. Under low-temperature and the combination of low-temperature and weak-light stress, the anthocyanin content increased due to the significant down-regulation of *3GT*.

## 1. Introduction

Eggplant (*Solanum melongena* L.), rich in anthocyanins, proteins, fats, carbohydrates, vitamins, and other substances, possesses high nutritional value and enjoys great popularity among consumers [1]. As one of the principal vegetable varieties cultivated in greenhouses, it frequently encounters issues of yield reduction and quality deterioration as a consequence of inadequate light intensity and low temperatures. Such low-temperature and weak-light conditions have emerged as significant impediments to the off-season cultivation of eggplants during the winter and spring seasons within protected facilities. Elucidating the effects of low temperatures and weak light on the growth, anthocyanin content and overall quality of eggplants is of crucial importance for precisely regulating the growth environment of eggplants and effectively resolving the environmental challenges that arise during the production process. Anthocyanins, alternatively referred to as anthocyanidins, constitute a class of natural water-soluble pigments capable of endowing plants with a spectrum of colors, including purple, lilac, red, pink, and blue, in which the intensity of the coloration varies in accordance with the pigment content [2]. The purple hue manifested by the eggplant peel predominantly stems from the presence of anthocyanins therein. As the accumulation of anthocyanins progresses, the peel color transitions from fuchsia to a darker shade of blackish purple.

Anthocyanins are synthesized through the phenylpropanoid pathway and the flavonoids’ biosynthetic pathway [3]. The anthocyanin synthesis pathway represents a crucial branch within the flavonoid synthesis pathway. It encompasses a series of intricate enzymatic reactions and can mainly be divided into three distinct stages: forming the fundamental anthocyanin skeleton, producing anthocyanin precursors, and modifying these precursors into diverse anthocyanin glycosides [4,5]. In the process of anthocyanin synthesis, phenylalanine is first converted into 4-coumaroyl-CoA under the action of phenylalanine ammonia-lyase (PAL), cinnamate 4-hydroxylase (C4H), and 4-coumarate-CoA (4CL). Then, 4-coumaroyl-CoA is synthesized into dihydroflavonol under the action of chalcone synthase (CHS), chalcone isomerase (CHI), and flavanone 3-hydroxylase (F3H). Subsequently, through three branches, dihydroflavonol is transformed into different types of anthocyanidins under the action of flavonoid 3′-hydroxylase (F3′H), flavonoid 3′,5′-hydroxylase (F3′5′H), dihydroflavonol 4-reductase (DFR), and anthocyanidin synthase (ANS), and further anthocyanins are formed. F3′H and F3′5′H determine the branching and types of anthocyanin synthesis. F3H converts naringenin into dihydrokaempferol (DHK). Subsequently, if catalyzed by F3′H, dihydroquercetin (DHQ) is generated and further catalyzed by *DFR*, *ANS*, and UDP-glucose/flavonoid 3-O-glucosyltransferase (UFGT) to produce cyanidin-type anthocyanins; if catalyzed by F3′5′H, dihydromyricetin (DHH) is produced and further catalyzed by DFR, ANS, and UFGT to yield delphinidin-type anthocyanins [6,7]. The enzymes related to the anthocyanin synthesis pathway are mainly encoded by structural genes. These structural genes can be classified into early biosynthetic genes, including *CHS*, *CHI*, *F3H*, and *F3′H*, and late biosynthetic genes, such as *F3′5′H*, *DFR*, *ANS*, and *UFGT* [8,9]. The expression of these structural genes is co-regulated by environmental factors and transcription factors (TFs). Among the TFs, myeloblastosis (*MYB*), basic helix–loop–helix (*bHLH*), and *WD40* are the core TFs, which regulate the transcription of structural genes in the form of the MBW (MYB-bHLH-WD repeat) complex [2,10,11,12,13].

The synthesis and accumulation of anthocyanins are influenced by environmental factors, with temperature and light playing particularly prominent roles. Temperature and light regulate anthocyanin synthesis by inducing and modulating the expression of upstream genes such as *PAL*, *CHS*, and *CHI*, as well as downstream genes *DFR* and *ANS* in the anthocyanin synthesis pathway [14,15]. Moreover, temperature and light can also regulate anthocyanin synthesis through the regulation of transcription factors like *MYB*, *PsbHLH1*, and *PsbHLH3* that are associated with anthocyanin synthesis. Under low-light conditions, *SmMYB1* in eggplants is up-regulated, while the structural genes for anthocyanin synthesis are down-regulated, leading to a decrease in anthocyanin content [9,16,17]. Zhou [18] found that when *SmTT8*, the *bHLH* partner of *SmMYB113*, was co-expressed with *SmCBF* and *SmMYB113*, the anthocyanin content increased significantly compared to that of *SmCBF* and *SmMYB113* alone. Additionally, the overexpression of *SmCBF2* and *SmCBF3* could facilitate the accumulation of anthocyanins in Arabidopsis under cold conditions. Yu et al. [19] discovered that the expression of *AcMYB10* in kiwifruit was highly correlated with the accumulation of natural pigment anthocyanins during fruit ripening, as well as the light- and temperature-induced pigment accumulation in callus. Furthermore, some scholars have indicated that *AP2/ERF* can regulate the synthesis of anthocyanins in plants [20]. Chen et al. [21] also found that the down-regulation of the *AP2/ERF* (*JcERF035*) gene might contribute to the biosynthesis and accumulation of anthocyanins in the aerial tissues of plants.

Transcriptome sequencing can simultaneously detect the expression levels of a large number of genes. By analyzing the gene expression levels, differential expression, and regulatory networks using transcriptome technology, we can provide technical methods for the identification and validation of candidate genes. Luo et al. [22] discovered 12 structural genes such as *CHS*, *CHI*, *F3H*, *DFR*, *ANS*, and *UFGT* and three transcription factors including *MYB113*, *GL3*, and *TTG2* when studying anthocyanin synthesis in eggplants using RNA-Seq technology, providing important clues for further research on the molecular mechanism of anthocyanin synthesis in eggplants. Zhang et al. [23] analyzed and determined the impact of high-temperature stress on the expression of genes in the anthocyanin biosynthesis pathway of eggplants using RNA-Seq technology. Moglia [24] combined RNA-Seq and qPCR techniques to reveal the specific transcriptional patterns of candidate genes related to anthocyanin biosynthesis in eggplants in different plant tissues, organs, and fruit development stages. He et al. [25] combined RNA-Seq technology with gene overexpression to verify the function of SmWRKY44 in anthocyanin biosynthesis in eggplants. This indicates that transcriptomic methods can provide abundant gene expression information, assist in the identification of candidate genes, and verify the functions of candidate genes through various technical means, making them effective technical tools in the identification and validation of candidate genes.

To understand the impacts of low temperature, weak light, and the combination of low temperature and weak light on the anthocyanin content in eggplants, differential gene analysis was carried out on the eggplant peels under the treatments of low temperature, weak light, and the combination of low temperature and weak light with the help of transcriptome technology. This was performed to further disclose the influence mechanisms of low temperature, weak light, and the combination of low temperature and weak light on the accumulation of anthocyanins in the peels of eggplant, providing new ideas for the autumn–winter production of eggplant and the research on anthocyanins.

## 2. Results

### 2.1. Anthocyanin Content in Different Parts of Eggplant at Seedling, Flowering, and Fruit Stages Under Low Temperature and Weak Light

**Figure 1:**
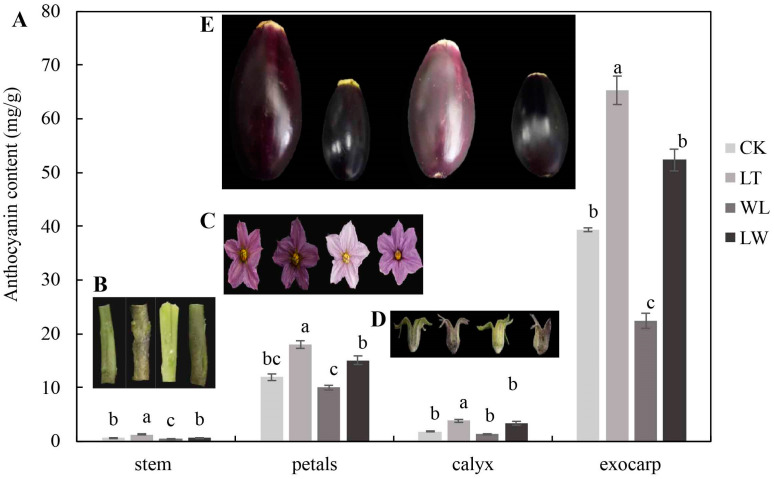
Anthocyanin contents in different parts of eggplant. (**A**) Anthocyanins contents in different parts of eggplant at seedling, flowering, and fruit stages under low temperature and weak light. (**B**) The color of seedling stem 8 d after treatment. (**C**) The color of eggplant petals at flowering stage under control (CK), low-temperature (LT), weak-light (WL), and low-temperature combined with weak-light (LW) conditions, respectively. (**D**) The color of eggplant calyx at flowering stage under CK, LT, WL, and LW conditions, respectively. (**E**) The color of eggplant fruit under CK, LT, WL, and LW conditions, respectively. From left to right, the sample in (**B**–**E**) are under CK, LT, WL, and LW conditions, respectively. Different lowercase letters in (**A**) indicate significant differences at *p* < 0.05.

**Figure 2:**
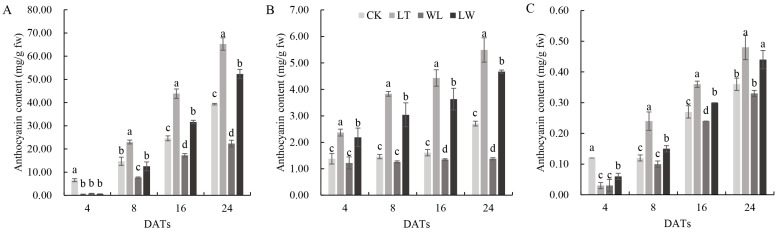
Anthocyanin content in different parts of the eggplant. (**A**) Anthocyanin contents in the eggplant peel. (**B**) Anthocyanin contents in the sarcocarp of eggplant. (**C**) Anthocyanin contents in the carpopodium of the eggplant. Different lowercase letters indicate significant differences at *p* < 0.05.

During the seedling stage, under the stresses of low temperature, weak light, and the combination of low temperature and weak light, the content of anthocyanin in the stem epidermis of eggplant seedlings reached the highest level. After 8 days of stress treatment (DAT, day after treatment), the content of anthocyanin in the stem epidermis was highest in low-temperature (LT) treatment, which was 82.14% higher than that in the control (CK), and was the least under weak light (WL), which was 29.93% lower than CK. There was no significant difference between the treatment of combined weak light and low temperature (LW) and CK (Figure 1). In the leaves and leaf veins of seedlings, the content of anthocyanin was highest under LT and lowest under LW (Table S1). During the flowering stage, the content of anthocyanin in the petals of the eggplant was highest at the full blooming stage (8 DATs). Among them, under LT stress, the content of anthocyanin was highest and the petal color was darkest (Figure 1). Under WL stress, the content of anthocyanin in the petals was lowest and the petal color was the lightest. The content of anthocyanin in the calyx was highest at the withering stage (Table S1). Under LT stress, the content of anthocyanin in the calyx was highest, being 77.11% higher than CK. Under WL and LW stress, the content of anthocyanin in the calyx had no significant difference with that in CK. During the fruiting stage, the contents of anthocyanin in the pericarp, pulp, and fruit stalk were all highest under LT treatment (Figure 2), and the content of anthocyanin in the peel was highest at 24 DATs (Figure 1). Under LT stress, the content of anthocyanin in the peel was maximally 65.86% higher than CK. Under WL stress, the content of anthocyanin in the peel of eggplant was lowest, being 43.21% lower than CK. This indicates that low temperature, weak light, and the combination of low temperature and weak light can significantly affect the content of anthocyanin in different parts of eggplant at different growth stages. Specifically, low temperature can significantly increase the content of anthocyanin in eggplant, while weak light can significantly reduce the content of anthocyanin in eggplant.

### 2.2. Analysis of Transcriptome in Eggplant Pericarp Under Low-Temperature and Weak-Light Stress

To clarify the underlying mechanism of anthocyanin biosynthesis in eggplant under low-temperature and weak-light stress, the exocarp of eggplant under low-temperature, weak-light and low-temperature–weak-light conditions were subjected to transcriptome sequencing. Twelve transcriptome databases, comprising control group (CK), low temperature (LW), weak light (WL) and low temperature–weak light (LW) with three replications of each group, were constructed.

A total of 60.62 million raw reads were obtained with an average of 8.54 Gb data for each sample. After filtering, 56.95 million clean reads were mapped to the *S. melongena* genome. The mapped percentage ranged from 94.84 to 95.38%, and the unique mapped percentage was about 92%. The GC contents of clean reads was about 43%. The average Q30 of clean reads was about 90.01% and ensured the high quality of sequence data and subsequent analysis (Table 1). We identified 31,271 genes, of which 27,635 were known genes and 3536 were novel genes.

**Figure 3:**
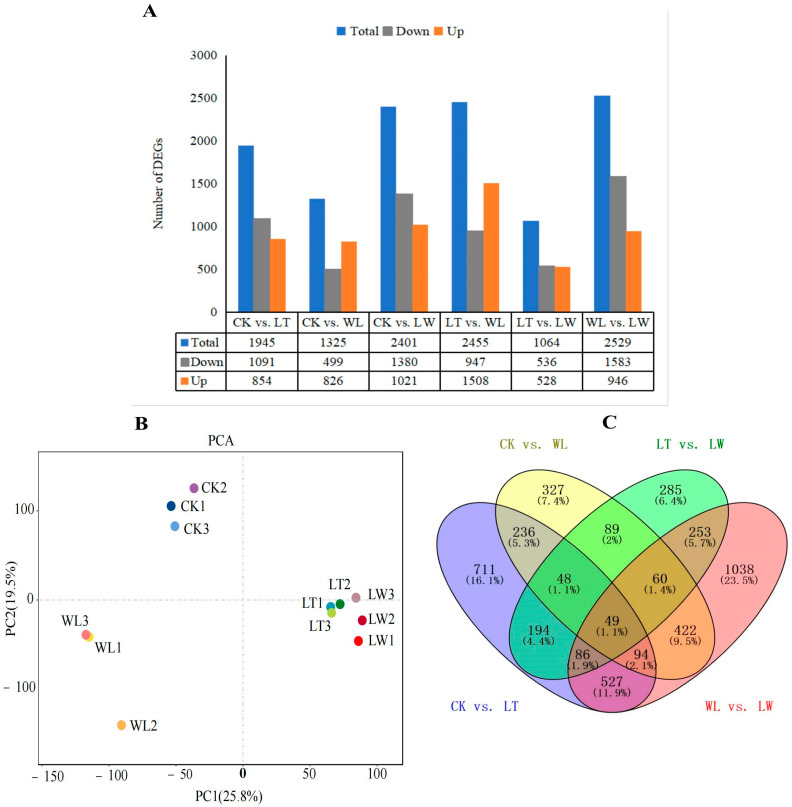
Qualification of RNA sequencing data in eggplant pericarp under low-temperature and weak-light stress. (**A**) Histogram of the numbers of differentially expressed genes (DEGs) identified in the pericarp of CK, LW, WL, and LW groups. (**B**) PCA of different samples used for RNA sequencing. CK, LW, WL, and LW denote the eggplant growth under normal temperature and light, low-temperature and normal light, normal temperature and weak-light, low-temperature and weak-light conditions, respectively. Each group contains three replicates. (**C**) Venn diagram showing the number of DEGs between differential groups in CK vs. LT, CK vs. WL, LT vs. LW, and WL vs. LW.

Next, we analyzed the correlation of sequenced samples and the distribution of gene expression (Figure 3). Principal component analysis (PCA) was performed to evaluate the similarity of the 12 complementary deoxyribonucleic acid (cDNA) databases. The three replications of each of the four groups (CK, LT, WL, and LW) clustered together, and gene expression was strongly correlated within each group (Figure 3B). The differentially expressed genes (DEGs) in the four groups were counted and are presented in Figure 3A. Compared to the control group (CK), 1945 and 1325 genes were differentially expressed in LT and WL groups, respectively, among which 854/826 genes were up-regulated and 1904/455 genes were down-regulated (Figure 3A). A total of 1064 DEGs between LT and LW were identified, of which 528 genes were up-regulated and 536 genes were down-regulated (Figure 3A). A total of 2529 DEGs between LW and WL were identified, of which 946 genes were up-regulated and 1583 genes were down-regulated (Figure 3A). There were 711, 327, 285, and 1038 specific DEGs in the compared groups of CK vs. LT, CK vs. WL, LT vs. LW, and WL vs. LW, respectively. A total of 49 DEGs common to the four groups were identified (Figure 3C).

### 2.3. Analysis of Differentially Expressed Genes in Eggplant Pericarp Under Low-Temperature and Weak-Light Stress

**Figure 4:**
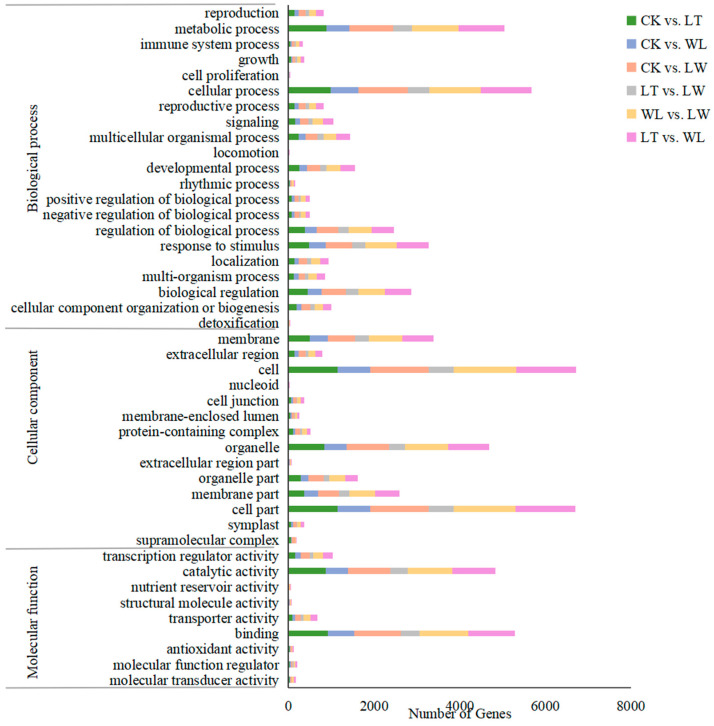
GO classification of differentially expressed genes (DEGs). The X axis represents the number of genes annotated to the GO terms, and the Y axis represents the classification of GO.

To identify the function of the DEGs, functional classification was performed based on annotations in the Gene Ontology (GO) database (Figure 4 and Figure S1). In total, 11,719 genes were assigned to the biological process (BP), cellular component (CC), and molecular function (MF) categories, which were enriched in 21, 14, and 9 terms, respectively (Figure 4). In CK vs. LT, CK vs. WL, CK vs. LW, LT vs. LW, WL vs. LW, and LT vs. WL comparisons, the DEGs exhibited a similar GO classification.

For all six groups, the three most highly enriched subcategories of BP were cellular process, metabolic process, and response to stimulus. Regarding CC, the most highly enriched subcategories for all six groups were cell, cell part, and organelle. In case of MF, the terms with the highest enrichment were binding, catalytic activity, and transcription regulator activity (Figure 4).

**Figure 5:**
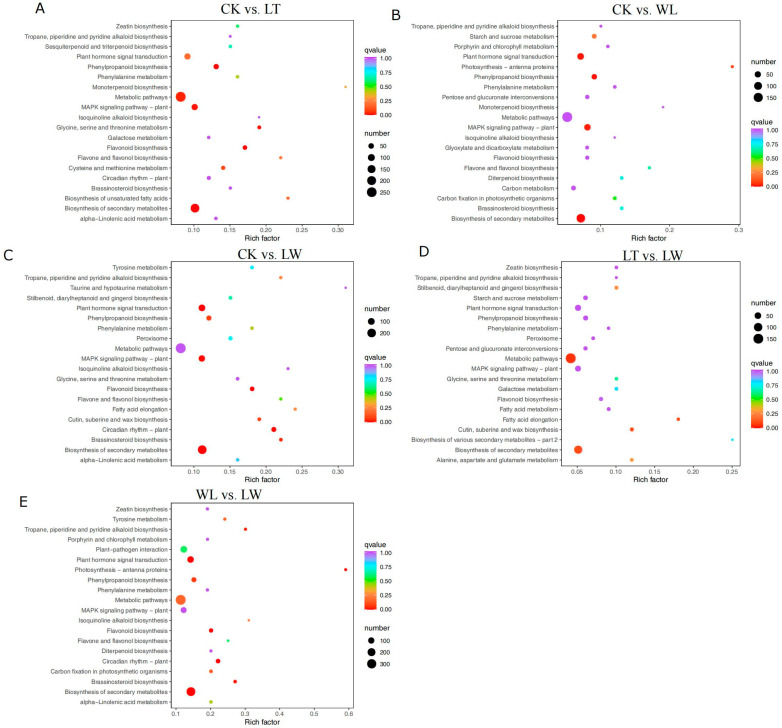
KEGG enrichment of DEGs in CK vs. LT (**A**), CK vs. WL (**B**), CK vs. LW (**C**), LT vs. LW (**D**), and WL vs. LW (**E**). The X axis is the Rich Factor (Rich Factor is calculated as candidate gene number in a specific term/total gene numbers) and the Y axis represents KEGG pathway; the size of the bubble indicates the number of genes annotated to a KEGG pathway. The color represents the enriched Q-value; color changes from purple to red represent the Q-value changes from big to small. The enrichment of the KEGG pathway is shown in the form of a bar chart, and the top 20 KEGG pathways are plotted with the smallest Q-value.

To explore the biological pathways in which the DEGs are involved, the Kyoto Gene and Genome Encyclopedia (KEGG) database was used for DEG classification [1]. A total of 6211 DEGs were assigned to 5 branches with 36 subbranches (Figure 5). After enrichment analysis, the 20 top-ranked pathways with the highest gene numbers and a low *p*-value were screened and are listed in Figure S1. In the CK vs. LT, CK vs. WL, CK vs. LW, LT vs. LW, WL vs. LW, and LT vs. WL comparisons, the pathway with the highest number of enriched DEGs was metabolic pathway, followed by the biosynthesis of secondary metabolites.

### 2.4. Expression Pattern of Anthocyanin Biosynthesis-Related Genes in Eggplant Under Low Temperature and Weak Light

**Figure 6:**
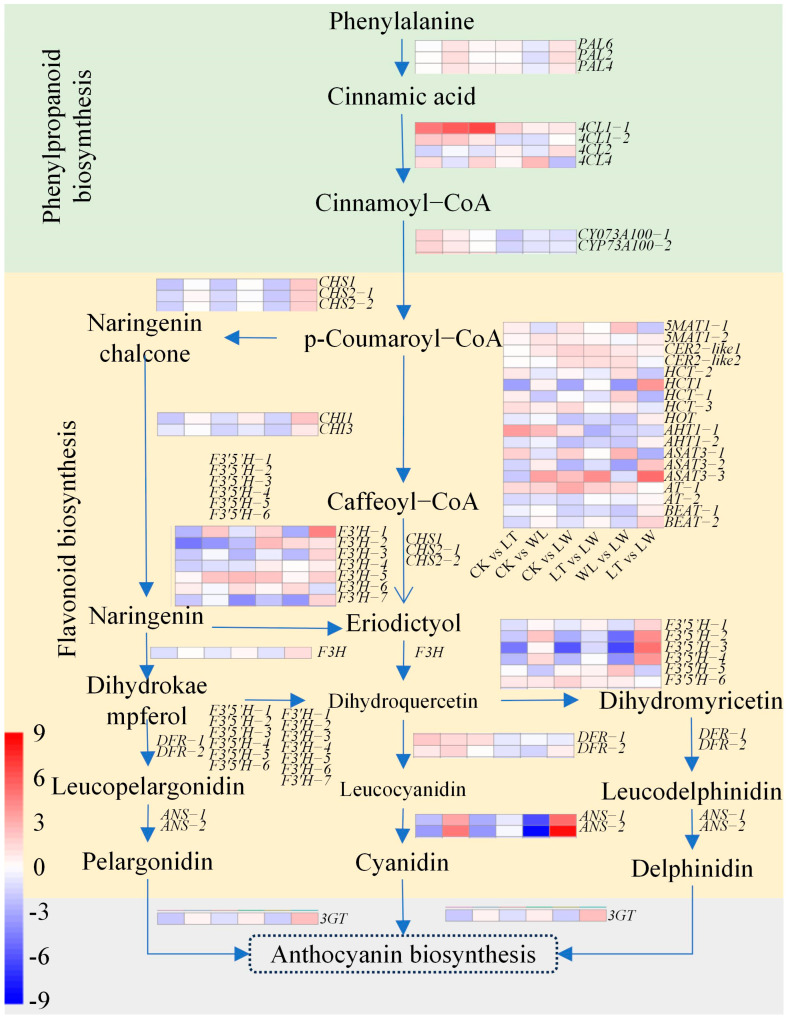
Comparative analysis of gene expression related to anthocyanin biosynthesis, phenylorpanoid biosynthesis, and flavonoid biosynthesis in eggplant under low temperature and weak light. The heatmap from left to right is the comparison between CK and LT, CK and WL, CK and LW, LT and LW, WL and LW, as well as LW and LW, respectively.

The biosynthesis of anthocyanins in eggplant involves three pathways: phenylorpanoid biosynthesis, flavonoid biosynthesis, and anthocyanin biosynthesis (Figure 6).

In the eggplant peel transcriptome, nine genes related to phenylorpanoid biosynthesis were identified (Figure 6). These genes include three *PAL* genes, *4CL* genes, and two *P450* homologous rans-cinnamate 4-monooxygenase (*CYP73A100*) genes (Figure 6). Forty-one genes related to flavonoid biosynthesis were identified (Figure 6). These genes contain three *CHS* genes, two *CHI* genes, eight shikimate O-hydroxycinnamoyltransferase genes, six *F3′5′H* genes, seven *F3′H* genes, one *F3H* gene, two *DFR* genes, and two *ANS* genes (Figure 6). In addition, there is one gene for anthocyanidin 3-O-glucosyltransferase (*3GT*), a key enzyme in the anthocyanin synthesis pathway.

In the genes of the eggplant peel, compared with the CK treatment, in the LT treatment, the genes *CYP73A100* (*Smechr0500036*, *Smechr0500037*), *4CL1* (*Smechr0700271*, *Smechr0501797*), *4CL4* (*Smechr0300074*), and *DFR* (*Smechr0400693*) were up-regulated, while the genes *4CL2* (*Smechr0302347*), *CHI1* (*Smechr1001863*), *CHS* (*Smechr0902497*, *Smechr0500402*, *Smechr0500409*), *F3H* (*Smechr0202240*), *F3′H* (*Smechr0601880*, *Smechr0802226, Smechr1002511*, *Smechr0802224*), *F3′5′H* (*Smechr1201797*, *Smechr0402167*, *Smechr0700499*, *Smechr0700573*), and *ANS* (*Smechr1001697*, *Smechr1002343*) were down-regulated (Figure 6). In the LW treatment, the genes *PAL* (*Smechr0900256*, *Smechr0500713*, *Smechr0900255*), *4CL1* (*Smechr0700271*, *Smechr0501797*), two F3′H genes (*Smechr0601880*, *Smechr0601259*), and two *F3′5′H* genes (*Smechr0402167*, *Smechr0700573*) were up-regulated, while one *F3′H* gene (*Smechr0802224*) and one *F3′5′H* gene (*Smechr0902646*) were down-regulated. In the WL treatment, the genes *4CL* (*Smechr0700271*, *Smechr0300074*), two *F3′H* genes (*Smechr0601259*, *Smechr0302294*), and one *F3′5′H* gene (*Smechr0700573*) were up-regulated, while the genes *CHI* (*Smechr1001863, Smechr0500261*), *CHS* (*Smechr0902497, Smechr0500402*, *Smechr0500409*), *F3′H* (*Smechr1002511*, *Smechr0802225*), *F3′5′H* (*Smechr0402167*, *Smechr0700499*, *Smechr0700573*), and *ANS* (*Smechr1001697*, *Smechr1002343*) were down-regulated.

The *3GT* (*Smechr1002540*) gene was down-regulated in the LT and LW treatments, and there was no difference in its expression level between the LW treatment and the CK treatment. There were certain differences in the effects of the three treatments on the expression of genes related to anthocyanin synthesis in the eggplant peel. It thus indicates that low temperature promotes the accumulation of anthocyanin by up-regulating three key genes for anthocyanin synthesis, namely *CYP73A100*, *4CL1*/*4CL4*, and *DFR*. Low light affects the accumulation of anthocyanin by up-regulating three key genes for anthocyanin synthesis, namely *PAL*, *4CL1*, and *ANS.* And low temperature combined with low light promotes the accumulation of anthocyanin by up-regulating the *4CL1* and *4CL4* genes and negatively regulating genes such as *ANS*.

### 2.5. Identification of Transcriptional Factors Regulating Anthocyanin Biosynthesis in Eggplant Under Low Temperature and Weak Light

**Figure 7:**
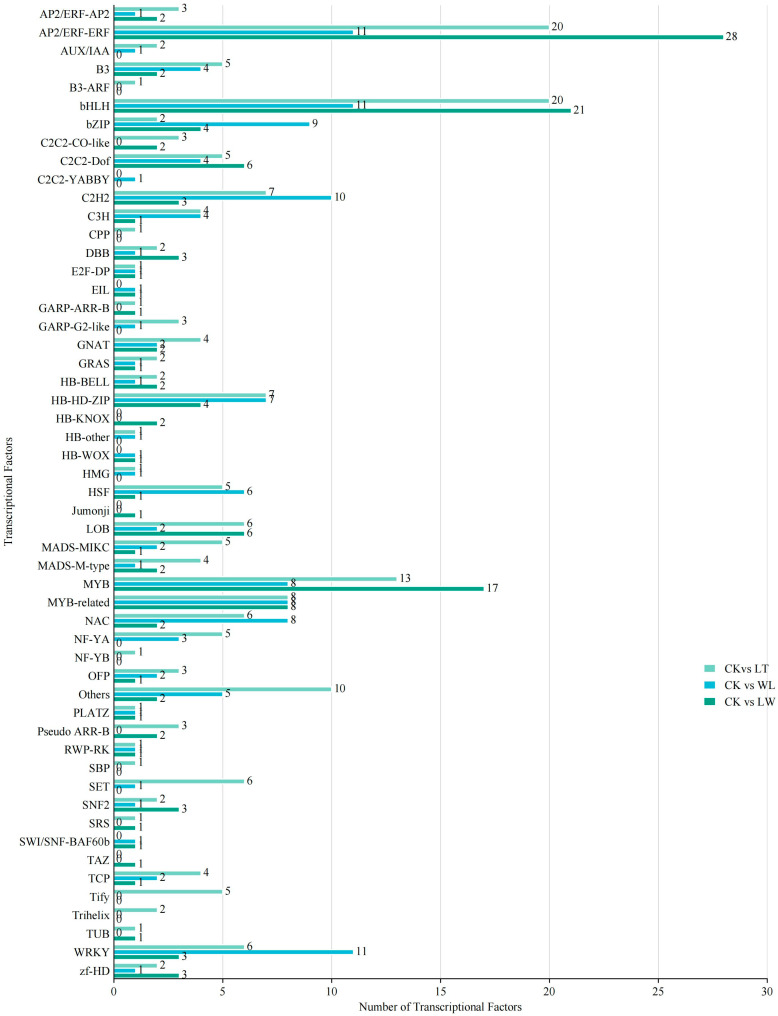
Numbers of transcriptional factors regulating anthocyanin biosynthesis in eggplant under low temperature and weak light.

Transcription factors were analyzed (Figure 7). Under LT stress, the top 3 transcription factor gene families were *AP2/ERF ERF* (a total of 20, with 18 down-regulated and 2 up-regulated), *bHLH* (a total of 20, with 16 down-regulated and 4 up-regulated), and *MYB* (13 in total, with 6 down-regulated and 7 up-regulated). Under WL stress, the top 3 transcription factor gene families were *AP2/ERF ERF* (a total of 11, with 3 down-regulated and 8 up-regulated), *bHLH* (a total of 11, with 4 down-regulated and 7 up-regulated), and *WRKY* (11 in total, with all 11 up-regulated). Under LW stress, the top 3 transcription factor gene families were *AP2/ERF ERF* (a total of 28, with 24 down-regulated and 4 up-regulated), *bHLH* (a total of 21, with 14 down-regulated and 7 up-regulated), and *MYB* (17 in total, with 8 down-regulated and 9 up-regulated).

It was found that transcription factors exist mainly in the form of large gene families, and most of them are transcription factors involved in the synthesis of secondary metabolites. Under the stresses of low temperature and the interaction between low temperature and low light, the top three transcription factor gene families are *AP2/ERF ERF*, *bHLH*, and *MYB*, indicating that these three transcription factor families play relatively important roles in responding to low temperature and the interaction between low temperature and low light. Interestingly, these three families are all related to anthocyanin synthesis, and most of the differential genes in the *MYB* family transcription factors belong to *R2R3 MYB*. Therefore, we speculate that *R2R3 MYB* is crucial for the synthesis of anthocyanin in peel of eggplant fruit under the stresses of low temperature, weak light, and the interaction between low temperature and weak light.

### 2.6. Real-Time qPCR of DEGs Related to Anthocyanin Biosynthesis in Eggplant

**Figure 8:**
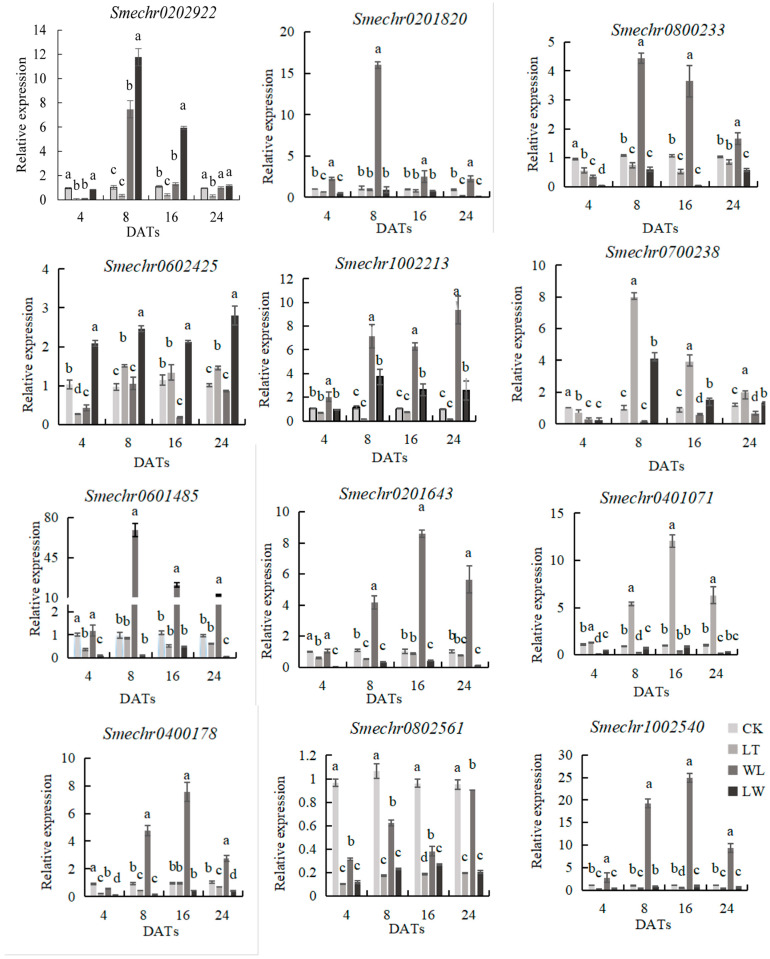
Real-time qPCR of DEGs related to anthocyanin biosynthesis in the eggplant peel. Different lowercase letters indicate significant differences at *p* < 0.05.

To verify and further analyze the expression profiles of genes related to anthocyanin synthesis in eggplants under low-temperature and low-light treatment, real-time qPCR (qRT-PCR) analysis was performed on relevant genes of eggplants at 2–8 DATs. As shown in Figure 8, the expression levels of all seven *MYB* transcription factors first increased and then decreased, with relatively high gene expression levels on the 8th and 16th days, indicating that a large amount of anthocyanin was accumulated during this stage. The genes *MYB1* (*Smechr1002213*) and *MYB106* (*Smechr0202922*) were up-regulated under WL and LW stresses but down-regulated under LT stress. The genes *MYB4* (*Smechr0601485*), *MYB102* (*Smechr0201820*), and *MYB6* (*Smechr0800233*) were down-regulated under LT and LW stresses and up-regulated under WL stress. The gene *MYB82* (*Smechr0700238*) was significantly up-regulated under LT stress, and the gene *MYB306* (*Smechr0602425*) was significantly up-regulated under LW stress. Among them, the expression trend of *MYB82* was consistent with that of anthocyanin content and was significantly up-regulated under LT stress, suggesting that the gene *MYB82* may be involved in anthocyanin biosynthesis and might be an important gene for anthocyanin synthesis under low-temperature stress.

The two *AP2/ERF-ERF* transcription factors showed a trend of first rising and then falling. The gene *AP2* (*Smechr0201643*) was significantly up-regulated under WL stress, with significant differences between WL and LT, as well as between WL and LW. The gene *ERF14* (*Smechr0401071*) was significantly up-regulated under LT stress, and its trend was consistent with that of anthocyanin content, indicating that the gene *ERF14* may be a key gene for anthocyanin synthesis under low-temperature stress.

The two *bHLH* transcription factors showed a trend of first rising and then falling. The expression level of the gene *bHLH105* (*Smechr0400178*) under each stress treatment on the 4th day was lower than that of the control (CK). On the 8th, 16th, and 24th days, it was significantly up-regulated under WL stress, with significant differences between WL and CK, between WL and LT, and between WL and LW. For the gene *bHLH14* (*Smechr0802561*), at the 4th, 8th, 16th, and 24th days, it was down-regulated, with significant differences between WL, LW, LT, and CK.

The gene *3GT* (*Smechr1002540*) in the anthocyanin biosynthesis pathway showed a trend of first rising and then falling and was significantly increased under WL stress. Its trend was consistent with that of anthocyanin content; this probably means that this is related to a decrease in anthocyanin levels in low light.

The trends of the 12 genes in Figure 8 on the 8th day were consistent with those of the transcriptome, indicating that the transcriptome information was reliable.

## 3. Discussion

In order to understand the mechanism through which the accumulation of anthocyanin content in diverse parts of eggplants is regulated by low temperature and weak light, we initially analyzed the anthocyanin content in various parts of eggplants subjected to low-temperature and weak-light treatments at different stages. Based on the differences in anthocyanin content, the outer epidermis of the eggplants during the fruiting stage was selected for transcriptome sequencing, aiming to analyze how low temperature and weak light regulate the anthocyanin content of eggplants. A total of 56 genes related to anthocyanin biosynthesis and 11 transcription factors were identified, and the expression of these genes under low-temperature and weak-light conditions was investigated. At each stage and for each part, the anthocyanin content was at its peak under low-temperature conditions and at its lowest under weak-light conditions, indicating that low temperature might enhance the accumulation of anthocyanin content, whereas weak light might suppress it.

### 3.1. Anthocyanin Content of Eggplant Under Low Temperature and Weak Light

After eggplant seedlings are subjected to low-temperature, weak-light, and combined low-temperature and weak-light stresses, the anthocyanin content in the stems is the highest. This may be related to the transportation of anthocyanins through the stems [26]. Under low-temperature and combined low-temperature and weak-light stresses, the stems of eggplant seedlings turn purple, while under weak-light stress, the stems are yellow-green (Figure 1B). Therefore, in production, the color of the stem epidermis can be used as a basis for judging whether eggplant seedlings are under low-temperature, weak-light, or combined low-temperature and weak-light stresses. During the flowering period, under low-temperature and combined low-temperature and weak-light stresses, the flower color of eggplants is purple, while under weak-light stress, the flower color is light pink (Figure 1C). Under low-temperature, weak-light, and combined low-temperature and weak-light stresses, the color difference in the peel is the most significant. Under low-temperature and combined low-temperature and weak-light stresses, the peel is black-purple, while under weak-light stress, the peel is purplish red (Figure 1E). This may be because low-temperature stress can increase the anthocyanin content in plant leaves, stems, flowers, and other parts [27,28], and also enhance the plant’s ability to biosynthesize anthocyanins [29]. Sufficient light can promote the accumulation of anthocyanin content [30], while insufficient light will inhibit the accumulation of anthocyanin content [31,32,33].

### 3.2. Regulation of Anthocyanin Biosynthesis of Eggplant Under Low Temperature and Weak Light

Under the stresses of low temperature, low light, and the interaction between low temperature and low light, the DEGs in the transcriptome were mainly enriched in biological processes such as cellular process, metabolic process, and response to stimulus based on GO functional annotation. The results of KEGG biological pathway classification and enrichment analysis of DEGs showed that there were the most differential genes in the metabolic and environmental information processing categories. Significantly enriched pathways included biosynthesis of secondary metabolites and phenylpropanoid biosynthesis, and both of these pathways are related to anthocyanin synthesis.

Studies have shown that *AP2/ERF* can regulate the biosynthesis of anthocyanins in plants [20,33]. Chen, Y. B. et al. [21] found that the down-regulation of the *AP2/ERF* (JcERF035) gene may contribute to the biosynthesis and accumulation of anthocyanins in the aerial tissues of plants. *MYB* transcription factors are involved in multiple metabolic pathways, such as secondary metabolism [34], growth and development [35], signal transduction [36], and disease resistance [37]. Yu et al. [19] found that the expression of *AcMYB10* is highly correlated with the accumulation of anthocyanins in natural pigmentation during fruit ripening and light/temperature-induced pigmentation in calli. Research on *bHLH* transcription factors mainly focuses on plant resistance research and the fields of plant biosynthesis and signal transduction. Among them, the regulation of anthocyanin synthesis by *bHLH* is one of its important functions [38,39]. Zhao et al. [40] showed that the overexpression of the *CpbHLH1* gene in model plants, Arabidopsis thaliana and tobacco, led to a sharp decline in anthocyanin content. The results of this study show that the anthocyanin content in the eggplant peel increases under the stresses of low temperature and the interaction between low temperature and low light, and decreases under low-light stress. In this study, under the LT treatment, three *4CL* genes, two *CYP73A100* genes, and an *AP2/ERF* genes were up-regulated, while *bHLH* genes and three *MYB* genes were down-regulated. Under the WL treatment, *PAL*, *4CL*, *bHLH* and *MYB* genes were up-regulated. This indicates that low temperature may promote the synthesis and accumulation of 4-coumaroyl-CoA by up-regulating the expression of *4CL* and *CYP73A100* through up-regulating *AP2/ERF* and down-regulating *bHLH* and *MYB*, and thus promote the synthesis and accumulation of anthocyanins by increasing the content of anthocyanin precursors. In contrast, weak-light treatment may inhibit the synthesis and accumulation of anthocyanins by up-regulating the expression of *bHLH* to regulate the structural genes related to anthocyanin synthesis. This suggests that the *AP2/ERF-ERF* transcription factor may play a positive regulatory role in anthocyanin biosynthesis, while the *bHLH* transcription factor may play a negative regulatory role. The biological functions of the *MYB* family of transcription factors are more complex, with different responses to various stresses, and further research is needed to clarify their regulation of anthocyanins biosynthesis.

## 4. Materials and Methods

### 4.1. Experimental Materials

The eggplant variety ‘Xiuniang’ was chosen as the test material, and its seeds were procured from Shandong Luyou Seedling Company. The “Xiuniang” variety exhibits the following characteristics: The fruits are long-ovoid in shape, with a rounded tip. At the optimal harvest time, the average single-fruit weight is around 170 g, the longitudinal diameter of the fruit is approximately 20 cm, and the transverse diameter is about 5 cm. The calyx is green, the fruit color is jet-black and lustrous, and the flesh is green. Initially, eggplant seeds of consistent size were selected, soaked for germination, and then sown in 50-hole seedling trays for raising seedlings. When the seedlings grew to the stage of having two leaves and one bud, those designated for the seedling stage experiment were transplanted into small flowerpots with a diameter of 8 cm and a depth of 6 cm, while those for the flowering- and fruiting-stage experiments were transplanted into cultivation bags with a diameter of 40 cm and a depth of 30 cm for further cultivation and standby. The seedling-raising and -cultivation substrates were all mixed substrates with a ratio of peat/vermiculite/perlite being 3:1:1. The diurnal temperature in the culture room was maintained at (25 ± 1) °C during the day and (18 ± 1) °C at night, the photoperiod was 12 h of light and 12 h of darkness, and the light intensity was 250 μmol/(m^2^·s). Treatments were commenced when the seedlings reached the stage of three leaves and one bud, after bud emergence, and 1 day after flowering and pollination, respectively.

### 4.2. Experimental Methods

In this experiment, four treatments were set up: low temperature (LT, 18/13 °C, 250 μmol/(m^2^·s)), weak light intensity (WL, 25/20 °C, 120 μmol/(m^2^·s)), low temperature combined with weak light intensity (LW, 18/13 °C, 120 μmol/(m^2^·s)), and the control (CK, 25/20 °C, 250 μmol/(m^2^·s)). The experimental treatments were carried out during the seedling stage, flowering stage, and fruiting stage of eggplants, respectively.

During the seedling stage, when the eggplant seedlings had three leaves and one bud, they were placed in an artificial climate light incubator (model: MGC-450HP-2) to undergo low-temperature and low-light-intensity stress. Seedlings at 0, 2, 4, 6, and 8 days after treatment (DATs) were sampled to measure the contents of anthocyanins in their leaves, leaf veins, stems, and roots, as well as to examine the morphological, physiological, and biochemical indices of the seedlings.

During the flowering stage, low-temperature and low-light-intensity stress was initiated when the eggplant flowering buds emerged. The petals and sepals of the opposite eggplants were collected at the bud stage (days after bud DABs, 8 days after bud emergence), the full bloom stage (11 DABs), and the decline stage (14 DABs) to determine their anthocyanin contents.

During the fruiting stage, artificial pollination was conducted on the second day after the flowers at the second branch bloomed, and stress was initiated one day after pollination. The carpopodium, exocarp of the pericarp, and sarcocarp of the eggplant at 4, 8, 16, and 24 DATs were collected to measure their anthocyanin contents. RNA was extracted from exocarp samples using the FastPlant Total RNA Isolation Kit (RC401). Samples collected on the 8th day were selected for transcriptome sequencing. There were 3 replicates in total, resulting in 12 samples. The remaining samples were frozen with liquid nitrogen and stored at −80 °C. Each treatment was replicated three times.

### 4.3. Index Testing

(1) Determination of anthocyanin content: The extraction method referred to that described by Qin Yan [41]. Approximately 1 g each of the leaves, leaf veins, stems, and roots at the seedling stage, the petals, and sepals at the flowering stage, and the fruit stalks, eggplant skins, and fruit flesh at the fruiting stage were used. The extraction solvent was 0.1% hydrochloric acid–methanol solution, and the ratio of sample mass to solvent volume was set at 1:10 (*m*/*V*). Ultrasonic extraction was carried out for 1 h under light-proof conditions, followed by centrifugation at 4000 r/min for 5 min. The entire supernatant was collected. After repeating the extraction three times, the extracts were combined and evaporated under reduced pressure at 30 °C until the volume was reduced to 1 mL. The absorbance values of each solution at 530 nm and 700 nm were measured by the pH differential method [42]. The anthocyanin content was calculated according to the formula proposed by Fuleki [43] as follows:

C(mg·g^−1^) = ΔA × V × n × M/(ε × m × L)

In the formula, ΔA represents the difference in the optical density (OD) values of anthocyanins at the absorption wavelength of 530 nm when the pH of the buffer is 1.0 and 4.5, that is, ΔA = (A530–A700)pH1.0 − (A530–A700)pH4.5. Here, A700 uses distilled water as a reference to eliminate the influence caused by the turbidity of the sample solution. V refers to the total volume of the anthocyanin extract (mL); M is the dilution factor; ε is the extinction coefficient of Cy-3-Glu (cyanidin-3-glucoside), which is 29,600; n is the relative molecular mass of Cy-3-Glu, which is 449; m represents the sample mass (g); and L is the light path (cm).

(2) Transcriptome sequencing: The eggplant skins collected 8 days after treatment were pretreated by freezing with liquid nitrogen and then sent to Wuhan Metware Biotechnology Co., Ltd. (Wuhan, China) for RNA extraction, construction of cDNA library, sequencing, and alignment analysis. The eggplant genome information referred to for alignment is available at http://www.bioinformaticslab.cn/SolPGD (accessed on 25 May 2021).

(3) Quantitative fluorescence PCR: Samples from each treatment were collected, respectively, and RNA was extracted using the TIANGEN^®^ RNAprep pure Plant Kit (Bioer Lin, Fuzhou, China)with three replicates set for each. Subsequently, the RNA was reverse-transcribed into cDNA using the Hifair^®^ II 1st Strand cDNA Synthesis SuperMix for qPCR (gDNA digester plus) kit(Bioer Lin, Fuzhou, China) for subsequent use. Quantitative real-time polymerase chain reaction (qRT-PCR) was carried out using the Hieff^®^ qPCR SYBR^®^ Green Master Mix kit(Bioer Lin, Fuzhou, China). The primer sequences used are shown in Table 2.

High-throughput sequencing was performed through procedures including sample detection, library construction, library quality control, and machine sequencing. 

### 4.4. Statistical Analysis

The experimental data were analyzed by variance analysis using IBM SPSS Statistics 26.0 software, and multiple comparisons were conducted with the LSD method (*p* < 0.05). The sequencing data were aligned with the designated reference genome, and the expression levels of the obtained genes in different samples or different sample groups were analyzed in terms of differential expression analysis, functional annotation of differentially expressed genes, and functional enrichment analysis. The qRT-PCR data were analyzed by the 2^−ΔΔCT^ method. Excel 2013 software, Photoshop CS6 software, and Origin 2019 software were used for statistical analysis and chart drawing.

## 5. Conclusions

The anthocyanin content of each part of the eggplants was significantly affected by low temperature and weak light. The anthocyanin content of the eggplants increased under low-temperature stress, while the anthocyanin content of eggplants decreased under weak-light stress. Under low-temperature and weak-light stress, low temperature played a leading role, and the anthocyanin content of eggplants increased. Low temperature and low light affect the biosynthesis of anthocyanins in eggplants by regulating the expression of structural genes *CHS*, *CHI*, *F3H*, *DFR*, and *ANS*, and *3GT* by regulating *AP2/ERF-ERF*, *bHLH*, and *MYB* transcription factors.

## Figures and Tables

**Table 1:** Summary of RNA-Seq datasets from the peel of eggplant under low-temperature and weak-light stress.

Sample	Total Raw Reads (M)	Total Clean Reads (M)	Clean Base (G)	Mapped to Genome (%)	Unique Mapped (%)	Clean Reads Q30 (%)	Clean Reads’ GC Content (%)
CK1	69.68	65.95	9.89	95.38	92.25	90.68	43.09
CK2	48.78	45.78	6.87	95.09	92.16	89.7	43.16
CK3	64.04	58.07	8.71	94.98	91.88	89.99	43.09
LT1	56.33	53.49	8.02	95.01	91.94	89.91	42.86
LT2	68.61	64.29	9.64	95.14	92.06	90.45	42.9
LT3	69.25	64.30	9.64	94.92	91.80	90.19	42.86
WL1	59.45	56.08	8.41	95.06	92.04	89.89	43.00
WL2	50.79	47.42	7.11	95.00	91.79	89.86	42.88
WL3	58.67	55.79	8.37	95.06	92.06	89.93	42.87
LW1	56.23	53.55	8.03	95.15	91.89	89.85	42.91
LW2	57.93	54.39	8.16	94.84	91.75	89.42	42.95
LW3	67.68	64.27	9.64	95.22	92.04	90.19	42.97

Note: The data in the table are based on the transcriptome sequencing data of the eggplant epidermis on 8 DAT. Here, CK (1, 2, 3), LT (1, 2, 3), WL (1, 2, 3), and LW (1, 2, 3) are the three replicate samples in the control group, LT treatment, WL treatment, and LW treatment groups, respectively.

**Table 2:** Primers and reaction conditions of RT-qPCR.

Genes	Product Length	Primer	Primer Sequence (5′-3′)	Tm
*Actin*	145	F	GTCGGAATGGGACAGAATG	60.61
		R	GTGCCTCAGTCAGGGAACAGGGT	58.22
*Smechr1002213*	101	F	TGCTTCGGATGAAGTGGATCT	60.14
		R	ACCAGCAATAAGTGACCATCTG	60.61
*Smechr0601485*	255	F	TTACCGGGACGAACAGAT	59.82
		R	GATGAAAGTTGTGGTGAGCT	60.04
*Smechr0202922*	213	F	ACGGCTAGTGAAAATGGGA	59.83
		R	CTTGTGGGTTACGGGGTC	59.83
*Smechr0201820*	190	F	GAGTTGTAGGCTTCGTTGGAC	58.93
		R	AGATCAAGAAGATCAAGGCG	55.25
*Smechr0700238*	201	F	TGGCTTGGAAGGTTTTCGC	60.01
		R	GAAGAACCAACAACATCTGCCAC	60.01
*Smechr0602425*	92	F	CTTCAAGCTCTTCTTGGAAATCG	60.18
		R	AAGCCTCCTTTCCCATTGTCC	60.25
*Smechr0800233*	89	F	CTTAAGCCGGGACTCAAG	60.18
		R	ACCATCGACTACCCAAAAG	59.96
*Smechr0201643*	162	F	TCTTCGTCCGTCACAGTCCATAG	59.76
		R	AGCCGTCTCAAACGTGCCTAG	60.13
*Smechr0401071*	129	F	CTGAAGCTGCTGCTAGAGCT	60.13
		R	GATCATTTCCAGCGCCT	58.64
*Smechr0400178*	114	F	AAGGACTAGAGCTGAATCATGC	59.45
		R	TCAAGAAGCGCTGCCAA	60.04
*Smechr0802561*	135	F	AGTCTGTATCACCAGCGCAG	59.83
		R	ACATAGGAGCCGGAACTGT	58.01
*Smechr1002540*	101	F	ACATTCCACCTGAAGTTACAGC	60
		R	GATTGGAGTACTAAAGGGCC	60.18

## Data Availability

Data will be made available on request.

## Supplementary Materials

The following supporting information can be downloaded at https://www.mdpi.com/article/10.3390/plants14030478/s1, Figure S1: GO analysis of DEGs; Table S1: Anthocyanin contents in different parts of eggplant at different stages.

## Abbreviations

DATs, days after treatment; DABs days after bud; LT, low temperature; WL, weak light; LW, low temperature and weak light; CK, control; PCA, principal component analysis; DEGs, differentially expressed genes; GO, Gene Ontology; BP, biological process; CC, cellular component; MF, molecular function; KEGG, Kyoto Gene and Genome Encyclopedia; PAL, phenylalanine ammonia-lyase; 4CL, 4-coumarate-CoA ligase; *CYP73A100*, rans-cinnamate 4-monooxygenase; CHS, chalcone synthase; CHI, chalcone isomerase; *F3′5′H*, flavonoid 3′,5′-hydroxylase; *F3′H*, flavonoid 3′-monooxygenase; F3H, flavanone 3-hydroxylase; DFR, dihydroflavonol 4-reductase; ANS, anthocyanidin synthase; 3GT, anthocyanidin 3-O-glucosyltransferase; MYB, myeloblastosis; bHLH, basic helix–loop–helix; DHK, dihydrokaempferol; DHQ, dihydroquercetin; TF, transcription factor; *UFGT*, UDP-glucose/flavonoid 3-O-glucosyltransferase; DHH, dihydromyricetin; cDNA, complementary deoxyribonucleic acid.
