# Supplementary material for: Comparative Transcriptomic Analyses of Anthocyanin Biosynthesis Genes in Eggplant Under Low Temperature and Weak Light
Source: Plants (Basel). 2025 Feb 6;14(3):478. doi: 10.3390/plants14030478 (PMC11819703; doi:10.3390/plants14030478)
Supplement: Supplementary file 1 [file plants-14-00478-s001.zip › plants-3431409-supplementary.pdf]

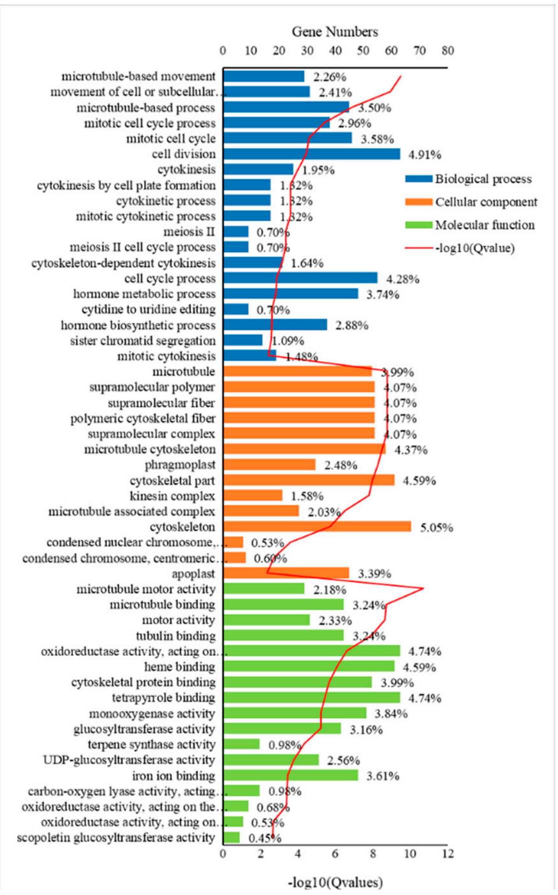
Gene Numbers
0 10 20 30 40 50 60 70 80
microtubule-based movement 2.26%
movement of cell or subcellular.. 2.41%
microtubule-based process 3.50%
mitotic cell cycle process 2.96%
mitotic cell cycle 3.58%
cell division 4.91%
cytokinesis 1.95%
cytokinesis by cell plate formation 1.32%
cytokinetic process 1.32%
mitotic cytokinetic process 1.32%
meiosis II 0.70%
meiosis II cell cycle process 0.70%
cytoskeleton-dependent cytokinesis 1.64%
cell cycle process 4.28%
hormone metabolic process 3.74%
cytidine to uridine editing 0.70%
hormone biosynthetic process 2.88%
sister chromatid segregation 1.09%
mitotic cytokinesis 1.48%
microtubule 3.99%
supramolecular polymer 4.07%
supramolecular fiber 4.07%
polymeric cytoskeletal fiber 4.07%
supramolecular complex 4.07%
microtubule cytoskeleton 4.37%
phragmoplast 2.48%
cytoskeletal part 4.59%
kinesin complex 1.58%
microtubule associated complex 2.03%
cytoskeleton 5.05%
condensed nuclear chromosome,.. 0.53%
condensed chromosome, centromeric.. 0.60%
apoplast 3.39%
microtubule motor activity 2.18%
microtubule binding 3.24%
motor activity 2.33%
tubulin binding 3.24%
oxidoreductase activity, acting on.. 4.74%
heme binding 4.59%
cytoskeletal protein binding 3.99%
tetrapyrrole binding 4.74%
monooxygenase activity 3.84%
glucosyltransferase activity 3.16%
terpene synthase activity 0.98%
UDP-glucosyltransferase activity 2.56%
iron ion binding 3.61%
carbon-oxygen lyase activity, acting.. 0.98%
oxidoreductase activity, acting on the.. 0.68%
oxidoreductase activity, acting on.. 0.53%
scopoletin glucosyltransferase activity 0.45%
Biological process
Cellular component
Molecular function
-log10(Qvalue)
0 2 4 6 8 10 12
-log10(Qvalues)

A

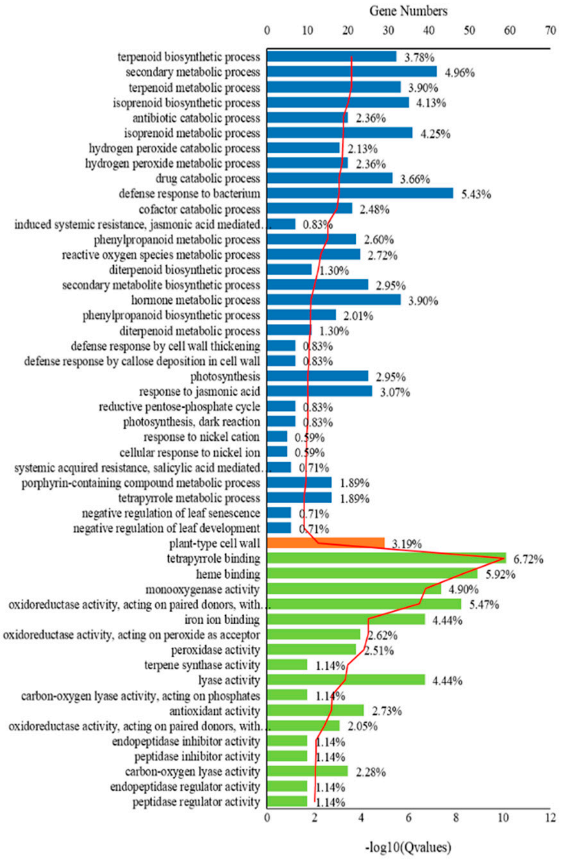
Gene Numbers
0 10 20 30 40 50 60 70
terpenoid biosynthetic process 3.78%
secondary metabolic process 4.96%
terpenoid metabolic process 3.90%
isoprenoid biosynthetic process 4.13%
antibiotic catabolic process 2.36%
isoprenoid metabolic process 4.25%
hydrogen peroxide catabolic process 2.13%
hydrogen peroxide metabolic process 2.36%
drug catabolic process 3.66%
defense response to bacterium 5.43%
cofactor catabolic process 2.48%
induced systemic resistance, jasmonic acid mediated.. 0.83%
phenylpropanoid metabolic process 2.60%
reactive oxygen species metabolic process 2.72%
diterpenoid biosynthetic process 1.30%
secondary metabolite biosynthetic process 2.95%
hormone metabolic process 3.90%
phenylpropanoid biosynthetic process 2.01%
diterpenoid metabolic process 1.30%
defense response by cell wall thickening 0.83%
defense response by callose deposition in cell wall 0.83%
photosynthesis 2.95%
response to jasmonic acid 3.07%
reductive pentose-phosphate cycle 0.83%
photosynthesis, dark reaction 0.83%
response to nickel cation 0.59%
cellular response to nickel ion 0.59%
systemic acquired resistance, salicylic acid mediated.. 0.71%
porphyrin-containing compound metabolic process 1.89%
tetrapyrrole metabolic process 1.89%
negative regulation of leaf senescence 0.71%
negative regulation of leaf development 0.71%
plant-type cell wall 3.19%
tetrapyrrole binding 6.72%
heme binding 5.92%
monooxygenase activity 4.90%
oxidoreductase activity, acting on paired donors, with.. 5.47%
iron ion binding 4.44%
oxidoreductase activity, acting on peroxide as acceptor 2.62%
peroxidase activity 2.51%
terpene synthase activity 1.14%
lyase activity 4.44%
carbon-oxygen lyase activity, acting on phosphates 1.14%
antioxidant activity 2.73%
oxidoreductase activity, acting on paired donors, with.. 2.05%
endopeptidase inhibitor activity 1.14%
peptidase inhibitor activity 1.14%
carbon-oxygen lyase activity 2.28%
endopeptidase regulator activity 1.14%
peptidase regulator activity 1.14%
0 2 4 6 8 10 12
-log10(Qvalues)

B

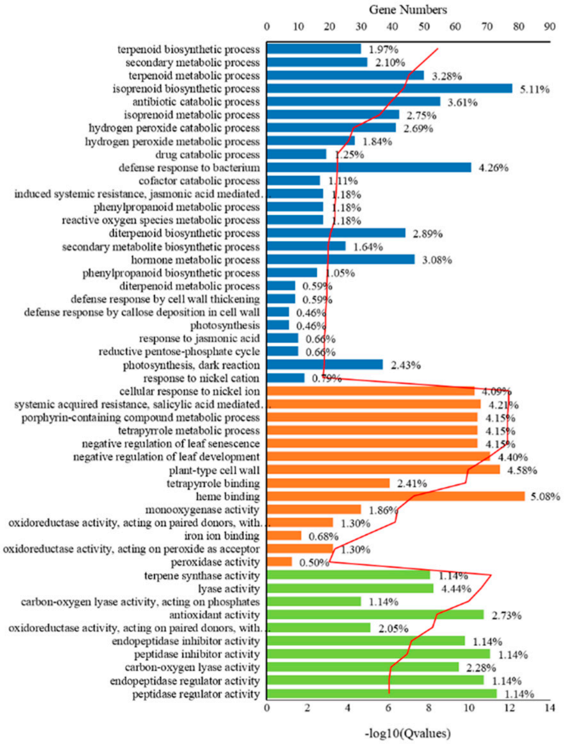
Gene Numbers
0 10 20 30 40 50 60 70 80 90
terpenoid biosynthetic process 1.97%
secondary metabolic process 2.10%
terpenoid metabolic process 3.28%
isoprenoid biosynthetic process 5.11%
antibiotic catabolic process 3.61%
isoprenoid metabolic process 2.75%
hydrogen peroxide catabolic process 2.69%
hydrogen peroxide metabolic process 1.84%
drug catabolic process 1.25%
defense response to bacterium 4.26%
cofactor catabolic process 1.11%
induced systemic resistance, jasmonic acid mediated.. 1.18%
phenylpropanoid metabolic process 1.18%
reactive oxygen species metabolic process 1.18%
diterpenoid biosynthetic process 2.89%
secondary metabolite biosynthetic process 1.64%
hormone metabolic process 3.08%
phenylpropanoid biosynthetic process 1.05%
diterpenoid metabolic process 0.59%
defense response by cell wall thickening 0.59%
defense response by callose deposition in cell wall 0.46%
photosynthesis 0.46%
response to jasmonic acid 0.66%
reductive pentose-phosphate cycle 0.66%
photosynthesis, dark reaction 2.43%
response to nickel cation 0.79%
cellular response to nickel ion 4.09%
systemic acquired resistance, salicylic acid mediated.. 4.21%
porphyrin-containing compound metabolic process 4.15%
tetrapyrrole metabolic process 4.15%
negative regulation of leaf senescence 4.15%
negative regulation of leaf development 4.40%
plant-type cell wall 4.58%
tetrapyrrole binding 2.41%
heme binding 5.08%
monooxygenase activity 1.86%
oxidoreductase activity, acting on paired donors, with.. 1.30%
iron ion binding 0.68%
oxidoreductase activity, acting on peroxide as acceptor 1.30%
peroxidase activity 0.50%
terpene synthase activity 1.14%
lyase activity 4.44%
carbon-oxygen lyase activity, acting on phosphates 1.14%
antioxidant activity 2.73%
oxidoreductase activity, acting on paired donors, with.. 2.05%
endopeptidase inhibitor activity 1.14%
peptidase inhibitor activity 1.14%
carbon-oxygen lyase activity 2.28%
endopeptidase regulator activity 1.14%
peptidase regulator activity 1.14%
0 2 4 6 8 10 12 14
-log10(Qvalues)

C

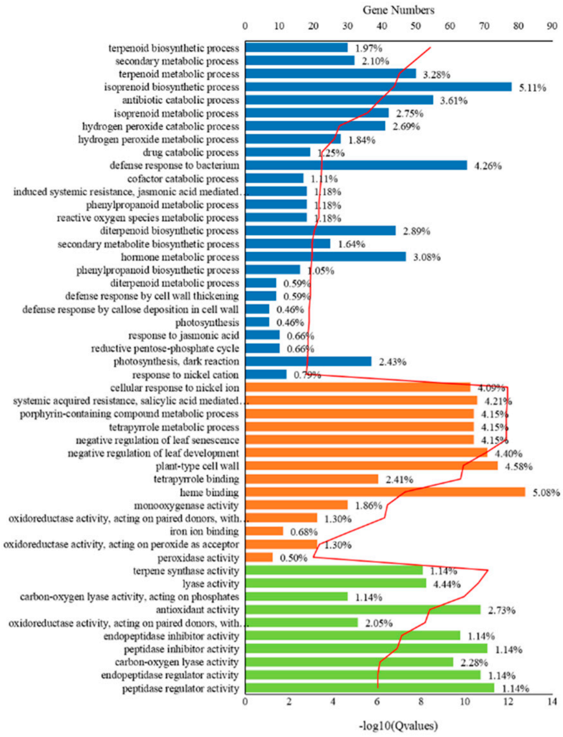
Gene Numbers
0 10 20 30 40 50 60 70 80 90
terpenoid biosynthetic process 1.97%
secondary metabolic process 2.10%
terpenoid metabolic process 3.28%
isoprenoid biosynthetic process 5.11%
antibiotic catabolic process 3.61%
isoprenoid metabolic process 2.75%
hydrogen peroxide catabolic process 2.69%
hydrogen peroxide metabolic process 1.84%
drug catabolic process 1.25%
defense response to bacterium 4.26%
cofactor catabolic process 1.11%
induced systemic resistance, jasmonic acid mediated.. 1.18%
phenylpropanoid metabolic process 1.18%
reactive oxygen species metabolic process 1.18%
diterpenoid biosynthetic process 2.89%
secondary metabolite biosynthetic process 1.64%
hormone metabolic process 3.08%
phenylpropanoid biosynthetic process 1.05%
diterpenoid metabolic process 0.59%
defense response by cell wall thickening 0.59%
defense response by callose deposition in cell wall 0.46%
photosynthesis 0.46%
response to jasmonic acid 0.66%
reductive pentose-phosphate cycle 0.66%
photosynthesis, dark reaction 2.43%
response to nickel cation 0.79%
cellular response to nickel ion 4.09%
systemic acquired resistance, salicylic acid mediated.. 4.21%
porphyrin-containing compound metabolic process 4.15%
tetrapyrrole metabolic process 4.15%
negative regulation of leaf senescence 4.15%
negative regulation of leaf development 4.40%
plant-type cell wall 4.58%
tetrapyrrole binding 2.41%
heme binding 5.08%
monooxygenase activity 1.86%
oxidoreductase activity, acting on paired donors, with.. 1.30%
iron ion binding 0.68%
oxidoreductase activity, acting on peroxide as acceptor 1.30%
peroxidase activity 0.50%
terpene synthase activity 1.14%
lyase activity 4.44%
carbon-oxygen lyase activity, acting on phosphates 1.14%
antioxidant activity 2.73%
oxidoreductase activity, acting on paired donors, with.. 2.05%
endopeptidase inhibitor activity 1.14%
peptidase inhibitor activity 1.14%
carbon-oxygen lyase activity 2.28%
endopeptidase regulator activity 1.14%
peptidase regulator activity 1.14%
0 2 4 6 8 10 12 14
-log10(Qvalues)

D

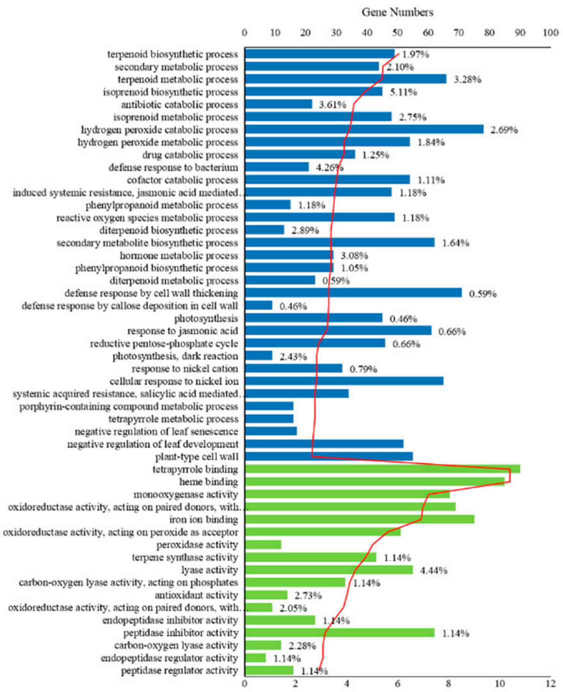

E

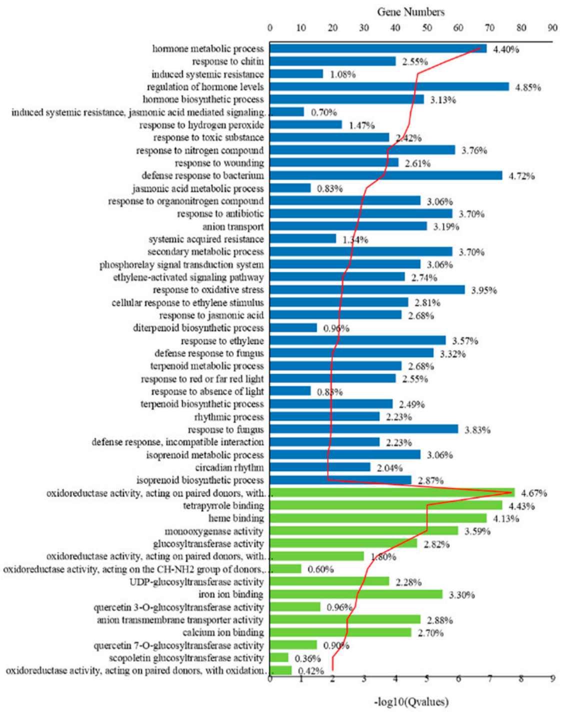

F

Figure S1. GO analysis of DEGs in CK vs. LT (A), CK vs. WL (B), CK vs. LW (C), LT vs. LW (D), WL vs. LW (E) and LT vs. WL(F). The upper X axis is the number of DEGs annotated to the GO term, and the lower X axis represents the -log10 (Q-value). The Y axis is the GO term. The enrichment of the GO term is shown in the form of a bar chart, and the top 20 GO terms are plotted with the smallest Q-value.

Table S1. Anthocyanin contents in different parts of the eggplant at different stages.

	**Anthocyanin contents of stem after 8 DATs**	**Anthocyanin contents of leaf after 8 DATs**	**Root after 8 DATs**	**Leaf Veins after 8 DATs**	**Petals in bud stage**	**Petals in fully open stage**	**Petals in wilting stage**	**Calyx in bud stage**	**Calyx in fully open stage**	**Calyx in wilting stage**	**Pericarp after 24 DATs**	**Carpopodium after 24 DATs**	**Sarcocarp after 24 DATs**
CK	0.72±0.01b	0.35±0.01b	0.14±0.01b	0.48±0.01b	4.86±0.15b	11.95±0.63c	10.87±0.67c	1.69±0.36c	1.83±0.16c	2.57±0.18b	39.35±0.01c	2.71±0.09b	0.36±0.02b
LT	1.31±0.05a	0.50±0.07a	0.17±0.02a	1.08±0.03a	6.83±0.60a	17.95±0.74a	14.87±0.86a	3.13±0.29a	3.85±0.25a	4.55±0.15a	65.26±0.01a	5.49±0.46a	0.48±0.04a
WL	0.51±0.06c	0.19±0.02c	0.1±0.01c	0.34±0.01c	3.52±0.53c	10.02±0.42d	7.71±0.46d	1.00±0.36d	1.34±0.02d	1.55±0.23c	22.34±0.01d	1.38±0.04b	0.33±0.01b
LW	0.73±0.02b	0.40±0.02b	0.14±0b	0.51±0.02b	6.01±0.79a	15.08±0.85b	13.45±0.3b	2.73±0.19b	3.36±0.36b	4.29±0.24a	52.33±0.01b	4.67±0.06a	0.44±0.03a
